# Handgrip strength as an indicator for death events in China: A longitudinal cohort study

**DOI:** 10.1371/journal.pone.0274832

**Published:** 2022-10-13

**Authors:** Kaihong Xie, Zhaojun Lu, Xiao Han, Meijia Huang, Junping Wang, Shou Kou, Weihao Wang, Sufang Zhuang, Weijun Zheng

**Affiliations:** 1 School of Nursing, Zhejiang Chinese Medical University, Hangzhou, China; 2 School of Public Health, Zhejiang Chinese Medical University, Hangzhou, China; 3 School of Health Humanities, Peking University, Beijing, China; 4 Office of Disciplinary Inspection and Supervision, The Third Affiliated Hospital of Zhejiang, Hangzhou, China; Korea University Medical Center, REPUBLIC OF KOREA

## Abstract

Studies have shown the indicative role of handgrip strength in health. However, there is limited evidence revealing its potential effect on death events among middle-aged and older adults in China. We aimed to prospectively evaluate if lower handgrip strength is associated with the event of death. Among 17,167 middle-aged and older adults between age 45 to 96, handgrip strength was collected by a handheld dynamometer in a Chinese longitudinal study of aging trend (CHARLS) 2011–2018. Using Cox proportional hazard models with exposures, we assessed the association between handgrip strength and death events. Elevated handgrip strength values were independently associated with the decreased death risk. These results illustrate that lower handgrip strength is an independent indicator of death risks among middle-aged and older Chinese, which highlights the significance of related intercessions. The median values of five levels of handgrip strength in the entire cohort were 16.5,23,28,33,42kg at baseline. A linear association existed between the handgrip strength values and the risk of all-cause death within 34.2kg. Handgrip strength can serve as an independent indicator for death risks.

## Introduction

Previous studies have explored the associations of distinct muscle-related factors with health outcomes [1]. Early adult life witnesses the peak of handgrip strength, which lasts until it finally declines with increasing age in people’s fifties [2]. The World Health Organization classifies people aged ≥45 as middle-aged and elderly. Grip strength begins to decline in middle age [3]. Handgrip strength predict health status in middle-aged and older adults [4]. In addition, some studies found that low handgrip strength was related with typical cerebrocardiovascular events, such as stroke, heart failure, and death caused by coronary heart diseases [5, 6]. Notably, handgrip strength was found to be inversely associated with cardiovascular disease morbidity [7–9], cancer mortality [10, 11], and death events [12, 13].

Future disability, morbidity, and mortality can be predicted by a basic but useful indicator, i.e., handgrip strength [14], which could be measured by applying scientifically reliable tools cost-effective for nationwide surveys; in addition, it is simple and non-invasive, yet revealing overall muscle strength [15] and thus used to diagnose sarcopenia and frailty across the lifespan [16].

A meta-analysis of 11 prospective cohort studies found that despite these lightweight associations, frail people with low muscular strength could face more risk to identify premature cancer mortality [17]. To date, the possibility of individual handgrip strength to predict incident mortality among middle-aged and older Chinese adults is still obscure, which provides for us the impetus to explore the correlation between handgrip strength and death events among Chinese middle-aged and older adults for a more convenient prediction of risk of death events. The purpose of this present study is to determine the predictive power of handgrip strength on all-cause death based on a nationally representative survey in China.

## Materials and methods

### Design and participants

**Fig 1:**
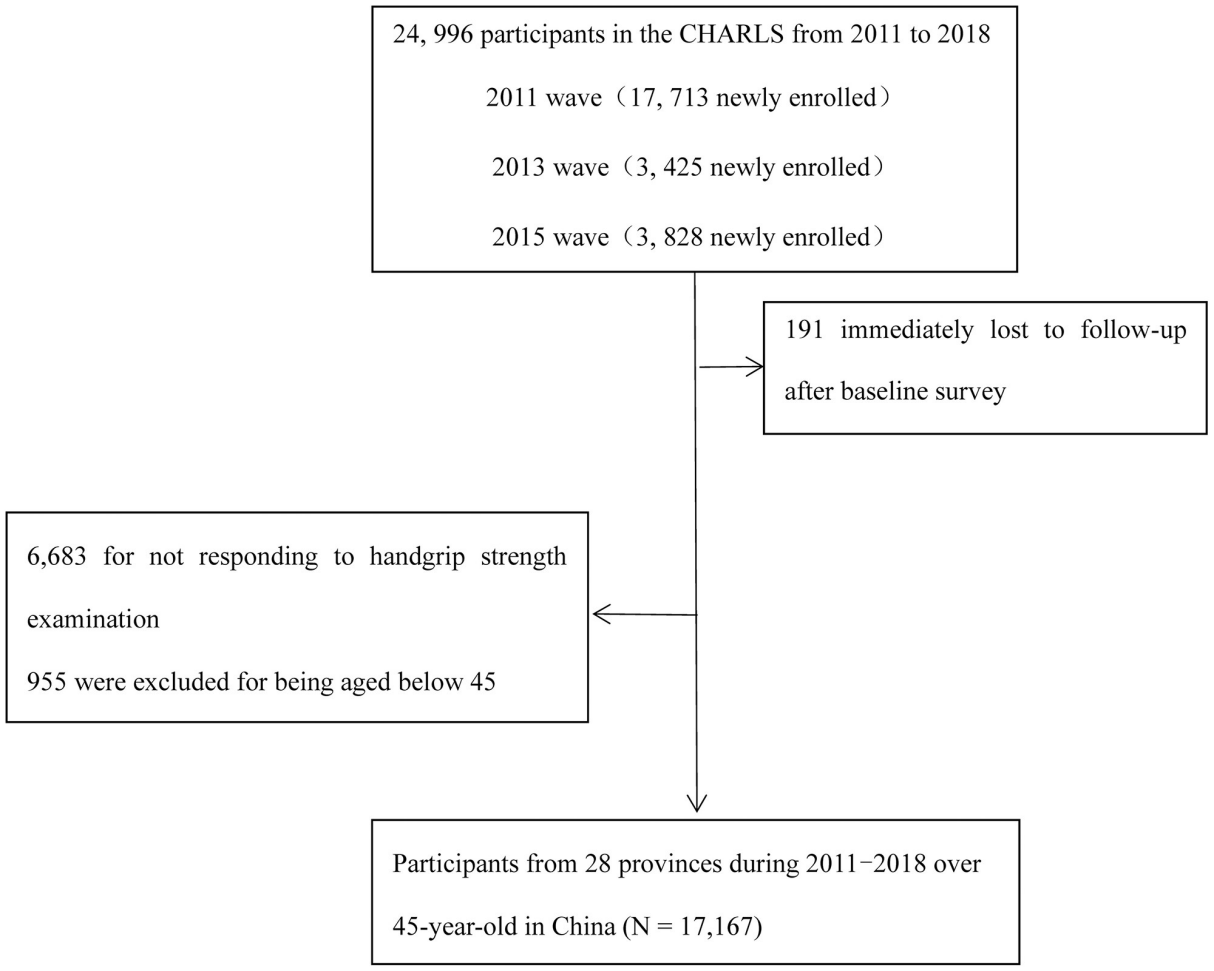
Flowchart of subject recruitment and eligibility.

The China Health and Retirement Longitudinal Study (CHARLS) selected a total of 24,805 cohort participants in 10,257 households from 150 counties/districts and 450 villages within 28 provinces, utilizing multistage stratified probability-proportional-to-size (PPS) sampling. The baseline survey was conducted in 2011 with new interviewees added in 2013 and 2015. Three follow-up surveys were carried out in 2013, 2015, and 2018. We excluded interviewees who did not respond to the handgrip strength test (n = 6,683) or were less than 45 years old in the four waves (n = 955). Thus, the final sample for analysis included 17,167 participants aged 45–96 years at baseline (Fig 1). All participants completed a standardized questionnaire to obtain sociodemographic features, lifestyles, and health-related behaviors and conditions. The response rate of the baseline survey (2011) was 73.1%. All participants accepted follow-ups every 2 to 3 years after the baseline survey. The CHARLS study was approved by the Institutional Review Board at Peking University Health Science Center (IRB number 00001052–11014). Written informed consent was appropriately signed by all participants before beginning the questionnaire.

### Handgrip strength measurement

Trained interviewer made sure that the physical examination would cause no harm to participants and participants reported no surgery, swelling, inflammation, severe pain, injury with one or both hands, or other past medical history warranting attention within the last 6 months. Handgrip strength were measured by asking participants to keeping squeezing the handle as hard as they could for several seconds before letting go, and then the measurement was repeated for their right and left hands separately in two alternative turns. A JAMAR dynamometer with an accuracy of 0.1 kg was employed for this physical examination. After the measurement, the dominant handgrip strength value was collected for the study. The values of handgrip strength were split into five categories per 5kg, using the median of the each group to represent five grip strength levels.

### Ascertainment of death events

CHARLS obtained death information from registrations and certifications by asking the deceased’s relatives or local communities in 2013, 2015, and 2018, or at the end of follow-up (March 31, 2019). The survival time of a respondent was defined as the length of time period ranging from accepting the first CHARLS survey to first record of death outcome, supposing no challenging credibility of previous death outcome from later data.

### Covariates

Covariates in this study were individual characteristics, encompassing demographic features, health behaviors and physical conditions [18]. Demographic features included age, sex, household registration, marital status, income, and education. Participants were classified as either married (official certificates were not necessary) or not (including but not limited to denial of marriage experience, not living together anymore, and divorce). Education was categorized by whether or not participants finished primary school, middle or senior high, or undergraduate [19]. Health-related behaviors included smoking and drinking defined as never, former, or current. Physical conditions were physician-diagnosed diseases (diabetes, hypertension, chronic lung disease, stroke, and heart disease), activities of daily living (ADLs), instrumental activities of daily living (IADLs), nighttime sleep or afternoon napping duration, physical functioning, and body mass index (BMI).

### Statistical analysis

For summary statistics, we employed means and standard deviation (SDs) to describe continuous variables conforming to the hypothesis of normal distribution, while medians and interquartile ranges were for nonnormally distributed continuous variables. Categorical variables were described by frequency with percentage. Based on baseline characteristics of handgrip strength, we deemed the χ^2^ test, analysis of variance, or Mann-Whitney U test as appropriate. Assuming missing at random, incomplete observations were imputed with multivariate imputation via classification or regression trees. Ten imputed data sets were generated and pooled using R 4.0.2.

To examine the association between handgrip strength and death events of all causes, Cox proportional hazards models were used to calculate hazard ratios (*HR*s) with 95% CIs. Proportional hazards assumption was justified for the participants (*P* = 0.224). We calculated the incidence of all-cause death with class interval set as 5 kg reduction in handgrip strength. Four models were estimated: in model 1, age and sex were adjusted; in model 2, BMI, marital status, educational level, marriage, and household registration were adjusted; in model 3, smoking and drinking were added. All 20 covariates were pooled in model 4.

To further examine the association between handgrip strength and death incidence of all causes, values of handgrip strength were split into five categories per 5kg reduction and then were included in Cox proportional hazards models with categories 1 as the reference group. Besides, we probed into any potential nonlinear relationship utilizing 5-knotted restricted spline regression. Subgroup analyses were intended for deciding whether the potential association between handgrip strength and death events was moderated by the following demographic and health characteristics: age, sex, household registration, marital status, education, smoking, drinking, falling down, sleep duration, hip fraction, income, diabetes, hypertension, chronic lung disease (doctor-diagnosed), stroke, heart disease, and BMI. *P* values for interaction were assessed with interaction terms and probability proportion tests. Two sensitivity analyses were designed as follows: (1) adjusting for fall down, hip fraction, ADL, physical function, sleep and nap time, incomes, diabetes, hypertension, chronic lung disease, stroke, and heart disease in model 4 in 17,167 participants; (2) repeating all analyses on ordinary data set (17,167 participants) without multiple imputations.

## Results

### Patient characteristics

A total of 17,167 participants were enrolled and accepted followed-up interviews to measure the risk factors (Fig 1). The age of participants at enrolment ranged from 45 to 96 years, and the median years of follow-up for the cohort was 7 years. Baseline characteristics of the study population were summarized in Table 1. The mean age of the population was 58.36 ± 9.9 years, and 8,872 (51.7%) participants were women. Participants were dominantly registered as agricultural household (12,526, 73.0%). During the follow-up, 1,453 death events had occurred. There were 1,787 participants with heart disease, 336 with stroke, and 1,591 participants had chronic lung diseases. As for behavioral risk factors, 5,336 (31.1%) participants reported current tobacco use, 4,446 (25.9%) reported current drinking alcohol. The mean score of physical function of the sample was 10.78 ± 4.8. Concerning metabolic risk factors, 3,667 (21.4%) had hypertension and 903 (5.3%) had diabetes. The mean BMI was 24.20 ± 13.03, and 10.05 ± 1.7 for ADL score.

**Table 1:** Baseline characteristics of 17,167 participants according to CHARLS.

		Dominant handgrip strength						
Total sample (*n* = 17,167)		<20(16.5), kg	20-25(23), kg	25-30(28), kg	30-35(33), kg	>35(42), kg	*p* ^a^	SMD
Baseline characteristics		*n* = 2,551	*n* = 2,684	*n* = 3,322	*n* = 2,758	*n* = 5,852		
Sex, *n* (%)								
	Man	395(15.5)	454(16.9)	937(28.2)	1,415(51.3)	5,085(86.9)	<0.001	0.902
	Female	2,154(84.5)	2,227(83.1)	2,384(71.8)	1,342(48.7)	765(13.1)		
Age, mean (SD)		64.66(11.03)	60.38(9.89)	58.57(9.80)	57.50(9.42)	55.00(8.05)	<0.001	0.456
BMI, mean (SD)		23.44(9.31)	24.60(21.69)	24.18(8.62)	23.96(7.54)	24.45(13.21)	0.008	0.047
Education level, *n* (%)							<0.001	0.477
	No formal education	1,711(70.8)	1,507(60.1)	1,599(51.4)	1,056(41.6)	1,411(26.5)		
	Primary school	388(16.0)	479(19.1)	691(22.2)	614(24.2)	1,346(25.3)		
	Middle or high school	306(12.7)	496(19.8)	762(24.5)	825(32.5)	2,389(44.9)		
	College or above	13(0.5)	27(1.1)	57(1.8)	45(1.8)	171(3.2)		
Married, *n* (%)							<0.001	0.249
	Yes	1,903(74.6)	2,247(83.7)	2,933(88.3)	2,463(89.3)	5,475(93.6)		
	No	648(25.4)	437(16.3)	389(11.7)	295(10.7)	377(6.4)		
Household, *n* (%)							<0.001	0.097
	Agricultural	2,006(84.1)	1,993(80.9)	2,486(80.5)	2,013(79.7)	4,028(76.7)		
	Non-agricultural	379(15.9)	471(19.1)	602(19.5)	514(20.3)	1,226(23.3)		
Smoking, *n* (%)							<0.001	0.516
	Never	2,042(80.1)	2,171(80.9)	2,464(74.2)	1,639(59.5)	1,982(33.9)		
	Formal	135(5.3)	125(4.7)	210(6.3)	246(8.9)	801(13.7)		
	Current	371(14.6)	386(14.4)	645(19.4)	871(31.6)	3,063(52.4)		
Drinking, *n* (%)							<0.001	0.444
	Never	2,136(83.8)	2,188(81.6)	2,573(77.5)	1,829(66.3)	2,537(43.4)		
	Formal	144(5.6)	174(6.5)	235(7.1)	235(8.5)	662(11.3)		
	Current	270(10.6)	320(11.9)	513(15.4)	694(25.2)	2,649(45.3)		
ADL, mean (SD)		13.01(6.00)	10.98(4.63)	10.36(4.20)	9.56(4.11)	8.41(3.84)	<0.001	0.45
Physical function, mean (SD)		14.11(5.85)	11.92(5.05)	10.98(4.42)	10.20(4.27)	8.97(3.53)	<0.001	0.507
Sleep, mean (SD)		6.03(2.14)	6.25(1.96)	6.34(1.86)	6.45(1.81)	6.59(1.64)	<0.001	0.138
Nap, mean (SD)		30.75(42.62)	30.62(43.14)	31.31(42.53)	33.22(42.61)	37.13(43.38)	<0.001	0.072
Fall down, *n* (%)							<0.001	0.142
	Yes	604(24.2)	508(19.1)	524(15.9)	412(15.0)	740(12.7)		
	No	1,891(75.8)	2,146(80.9)	2,773(84.1)	2,334(85.0)	5,080(87.3)		
Hip fraction, *n* (%)							<0.001	0.046
	Yes	66(2.6)	49(1.8)	54(1.6)	41(1.5)	73(1.3)		
	No	2,431(97.4)	2,603(98.2)	3,243(98.4)	2,706(98.5)	5,748(98.7)		
History of comorbidities								
Hypertension, *n* (%)		711(30.7)	607(25.5)	740(24.9)	555(23.1)	1,054(21.1)	<0.001	0.100
Chronic lung diseases, *n* (%)		308(13.3)	255(10.7)	315(10.6)	253(10.5)	460(9.2)	<0.001	0.092
Heart disease, *n* (%)		356(15.4)	334(14.1)	354(11.9)	296(12.3)	447(8.9)	<0.001	0.092
Diabetes, *n* (%)		171(7.4)	171(7.2)	183(6.2)	132(5.5)	246(4.9)	<0.001	0.055
Stroke, *n* (%)		80(3.4)	67(2.8)	70(2.3)	42(1.7)	77(1.5)	<0.001	0.064
Incomes, *n* (%)							<0.001	0.117
	Above average	67(3.3)	59(2.8)	68(2.6)	60(2.8)	134(3.1)		
	Average	1,010(49.1)	1,095(51.8)	1,371(52.1)	1,135(52.0)	2,321(53.6)		
	Relatively poor	607(29.5)	646(30.6)	829(31.5)	720(33.0)	1,421(32.8)		
	Poor	372(18.1)	313(14.8)	363(13.8)	266(12.2)	454(10.5)		

Abbreviation: SD, standard deviation.

^a^ P value was based on χ^2^ or analysis of variance or Mann-Whitney U test whenever appropriate.

^b^ Calculated as weight in kilograms divided by height in meters squared.

^c^ Measured in the subpopulation of 17,167 participants.

^d^ Dominant handgrip strength chooses five categories and takes the median. SMD, STD Mean Difference.

### Association of baseline handgrip strength and all-cause death

Table 2 shows the association between handgrip strength and death incidence of all causes. A linear and positive association between the handgrip strength values and the risk of death of all causes was found, i.e., greater handgrip strength was linearly associated with lower incidence of all-cause death.

**Table 2:** Incidence of death of all causes according to the handgrip strength states.

outcomes Death of all causes			HR (95%CI)			
Handgrip strength values, five categories (kg)	Cases, No.	Incidence Rate, per 1000 Person-Years	Model1^a^	Model2^b^	Model3^c^	Model4^d^
<20(16.5)	439	32.28	1 [Reference]	1 [Reference]	1 [Reference]	1 [Reference]
20-25(23)	230	15.54	0.62(0.52–0.73)	0.64(0.54–0.75)	0.63(0.53–0.74)	0.70(0.59–0.83)
25-30(28)	272	14.81	0.57(0.48–0.67)	0.60(0.51–0.71)	0.59(0.50–0.70)	0.68(0.57–0.81)
30-35(33)	199	12.94	0.46(0.38–0.55)	0.49(0.40–0.59)	0.48(0.40–0.58)	0.58(0.48–0.71)
>35(42)	313	9.71	0.39(0.32–0.47)	0.43(0.35–0.52)	0.42(0.35–0.51)	0.52(0.43–0.64)

Abbreviation: HR, hazard ratio.

^a^Model 1 was adjusted for age and sex.

^b^Model 2 was adjusted as model 1 plus educational level, marriage, and household registration.

^c^Model 3 was adjusted as model 2 plus BMI, smoking, and drinking.

^d^All 20 items were entered simultaneously in model 4.

### Nonlinear association between baseline handgrip strength and all-cause death

**Fig 2:**
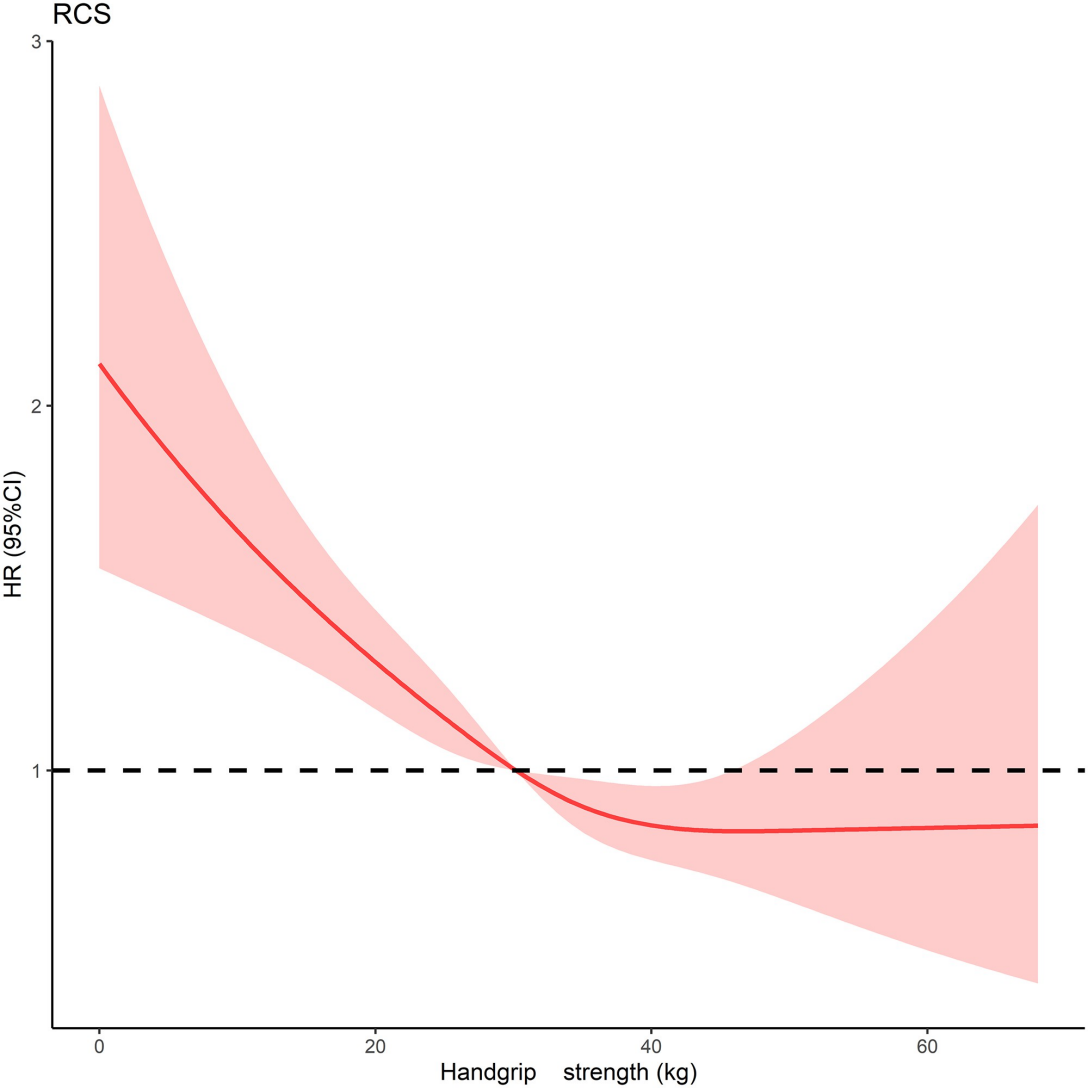
Adjusted Hazard Ratios (HRs) for death risks according to handgrip strength. Graphs show HRs for death of all causes adjusted for age, sex, BMI, household registration, marital status, education, income; smoking, drinking; and history of diabetes, hypertension, heart disease, stroke, and chronic lung disease; the history of falls, hip fraction, ADL, physical function, and sleep/nap duration. Data were fitted by a restricted spline Cox proportional hazards regression model.

Data were fitted by a restricted spline Cox proportional hazard regression model (Fig 2). A linear relationship existed between grip strength and death (for nonlinearity, *P* = 0.293). We selected the median grip strength as the reference point. The risk of all-cause death was relatively flat until around 34.2 kg of handgrip strength (*HR* = 0.92, 95%*CI*: 0.85–1.00).

### Association of handgrip strength and death risk stratified by covariates

**Fig 3:**
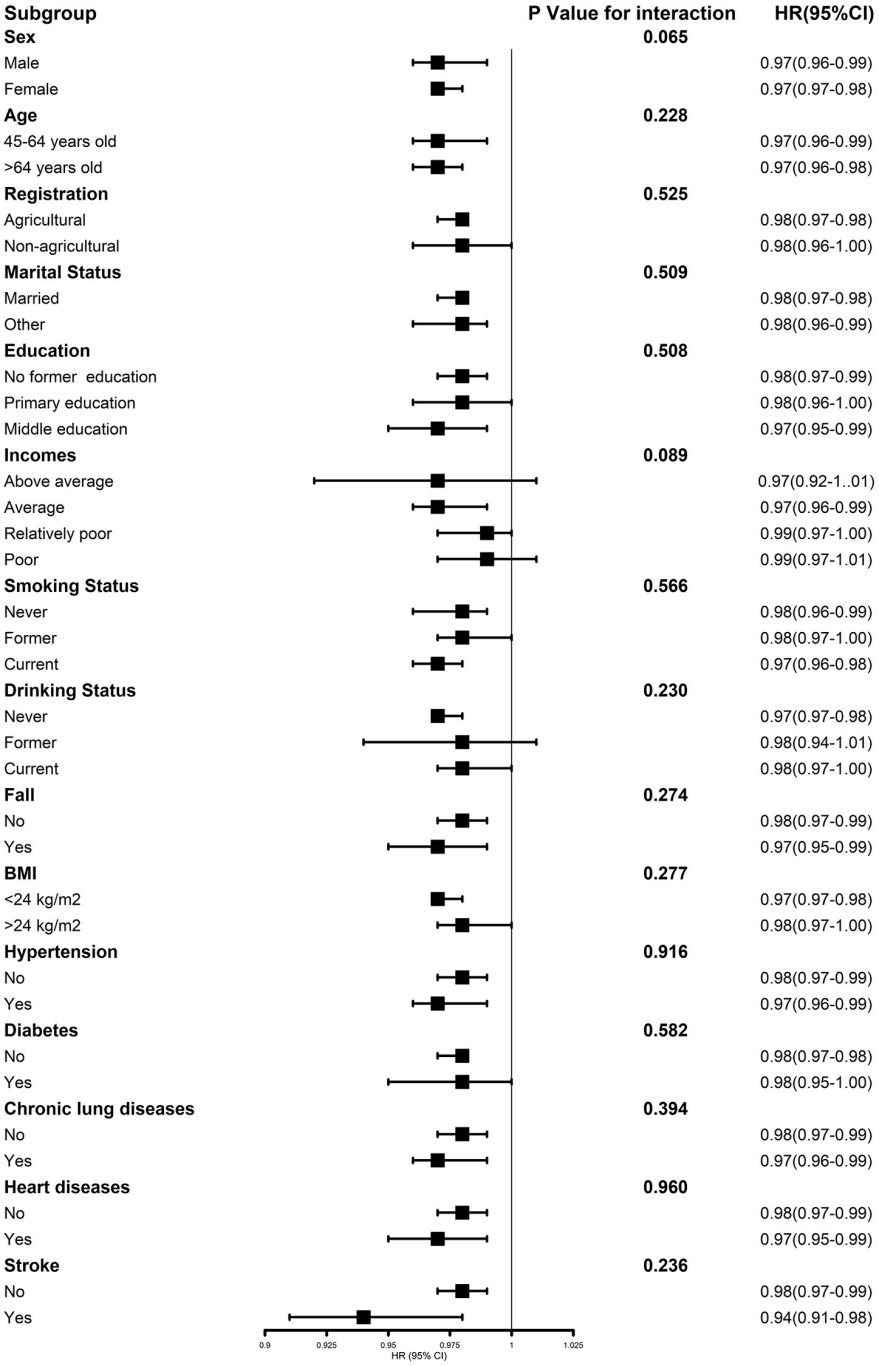
Association between handgrip strength values and death risk stratified by different factor. Graphs show hazard ratios (HRs) and 95% CIs for all-cause death after adjusting for all covariates.

Fig 3 shows the association between handgrip strength and death risks stratified by potential risk factors. There is no evidence for interaction between handgrip strength and death event of different factors. The results did not significantly change after adjusting for sex, age, registration, marriage, education, income, drinking/smoking status, BMI, falls, hypertension, diabetes, chronic lung disease, heart disease, and stroke. Similar patterns were found when analyses were repeated on original data as sensitivity test.

## Discussion

We found lower handgrip strength was linearly associated with higher incidence of death events in the nationwide longitudinal study of Chinese middle-aged and elderly followed over 8 years. The association was comparable over sex and age groups and were not completely accounted for by adjusting for sociodemographic, lifestyle-related, and health-related factors.

Our findings agree with findings from a meta-analysis of 42 prospective cohort studies including over 3 million participants [9]. One possible mechanism may be through endocrine system modulation. Skeletal muscle [19] has been recognized as a secretory organ that produces and expresses mytokines and peptides, such as IL-6 and brain-derived neurotrophic factor, in response to contraction. Mytokines can affect the regulation of glucose and lipid metabolism, contributing to the pathogenesis of obesity, diabetes, and other metabolic disorders. In addition, growth differentiation factor (GDF15) [1] is essential for optimal physical performance. Furthermore, myokines play a crucial role in counteracting harmful effects of proinflammatory adipokines, and peak flow of myokines was statistically significant in predicting mortality in both males and females [20] The relationship with all-cause death may be linear within a grip strength of 56 kg [9]. But in our study, a linear association was found within the grip strength of 34.2 kg, i.e., among people with lower grip strength. One possible reason may be adults over 45 years old in China suffer from early-childhood malnutrition [21]. Famine can cause malnutrition and deficiencies in body composition, which constituted an extreme loss of life [22]. In addition, Asians have significantly lower handgrip strength than Westerners [23].

Our findings are in line with previous studies in different countries such as Japan [24], Korea [12], Europe [25, 26], Russia [27], and America [28]. However, in patients over 91 years of age, there was no association between lower handgrip strength and change inability to walk, and no differences in the number of readmissions [29]. This may suggest that the predictive power of handgrip on the risk of death is limited in the oldest-old.

To the best of our knowledge, this is the first study to use a nationally representative and dynamic long-term follow-up cohort in the handgrip strength literature. We employed CHARLS data to illustrate the prognostic role of handgrip strength in the event of death among the Chinese middle-aged and older adults. There are several limitations. First, we investigated the cause of heterogeneity via stratification by several characteristics and prediction intervals [30]. However, subgroup analyses and p for interaction showed no evidence for heterogeneity. One explanation might be missing data in handgrip strength introduced bias because the most frail participants were excluded from the study. Second, our data for handgrip strength were cross-sectional and might neglect individual long-term trends. Third, all-cause death could capture sudden death events like traffic accidents and crimes, which may bring bias.

## Supporting information

S1 File Language assistant certificate. (PDF)

## Abbreviations

ADL: Activities of Daily Living
BMI: Body Mass Index
CI: Confidence Intervals
HR: Hazard Ratio
IADL: Instrumental Activities of Daily Living
