# Supplementary material for: Handgrip strength as an indicator for death events in China: A longitudinal cohort study
Source: PLoS One. 2022 Oct 13;17(10):e0274832. doi: 10.1371/journal.pone.0274832 (PMC9560503; doi:10.1371/journal.pone.0274832)
Supplement: S1 File — (PDF) [file pone.0274832.s001.pdf]

Language Assistant Certificate

This document is to certify that the article below has been edited by professional editors at AiMi (Scientific Editing Experts, United States. AiMi Academic Service, LLC) to ensure that the language is clear and free of errors. The intent of the author's message was not altered in anyway during the editing process. We guarantee the quality of our editing services, with the assumption that our suggested changes have been accepted and have not been further altered without the knowledge of our editors.

TITLE OF THE MANUSCRIPT

Handgrip strength as an indicator for death events in China: A longitudinal cohort study

AUTHORS

Kaihong Xie, Zhaojun Lu, Xiao Han, Meijia Huang, Junping Wang, Shou Kou, Weihao Wang, Sufang Zhuang, Weijun Zheng

AIMI ACADEMIC SERVICES SINCE 1984 AiMi Editor UNITED STATES Scientific Editing Experts

Kathleen Frey

Vice Chairman

Date: Jul. 11, 2022

AiMi, offers professional English language editing and publication support services to authors engaged in over 400 areas of research through its community of experienced editors, which includes doctors, published scientists, and researchers with peer review experience. Authors who work with AiMi are guaranteed excellent language quality and timely delivery.

Contact AiMi:
AiMi Academic Services, LLC
3338 Bradbury Rd, Madison, WI, 53719
www.aimieditor.com
inquiry@aimieditor.com
